# Direct Acting Antivirals in Hepatitis C-Infected Kidney Transplant Recipients: Associations with Long-term Graft Failure and Patient Mortality

**DOI:** 10.20411/pai.v5i1.369

**Published:** 2020-09-30

**Authors:** Michael R. Goetsch, Ashutosh Tamhane, Edgar T. Overton, Graham C. Towns, Ricardo A. Franco

**Affiliations:** 1 Department of Medicine, Johns Hopkins Hospital, Baltimore, Maryland; 2 Department of Medicine, Division of Infectious Diseases, University of Alabama at Birmingham, Birmingham, Alabama; 3 Department of Medicine, Division of Nephrology, University of Alabama School of Medicine, Birmingham, Alabama

**Keywords:** Direct-acting antivirals, kidney transplant, hepatitis C virus, proteinuria, mortality, graft failure, outcomes

## Abstract

**Background:**

Direct-acting antiviral (DAA) therapy among hepatitis C virus (HCV)-infected kidney transplant recipients is associated with short-term improvement in protein/creatinine (P/C) ratios, but how HCV cure affects long-term graft outcomes remains unknown.

**Methods:**

This is a retrospective follow-up study of 59 HCV-infected patients who underwent kidney transplant at the University of Alabama at Birmingham between 2007-2015 who were followed until the end of 2017. We examined the association of DAA-induced HCV cure with graft failure or death by survival analyses (Kaplan-Meier, Cox regression).

**Results:**

Mean age was 55 years, 73% were African American, and 68% were male. Median baseline creatinine was 1.4 mg/dL, P/C ratio was 0.5, and estimated glomerular filtration rate (eGFR) was 59 mL/min. Of those who received DAA, 24 (83%) achieved cure. The remaining 5 DAA patients (17%) did not have documented evidence of sustained virologic response (SVR). Overall, 19 (32%) patients experienced graft failure or death; with lower incidence in treated patients than untreated (4 vs 15 events; 2.6 vs 10.3 per 100 person-years [cHR 0.19, 95% CI: 0.06–0.66]). When adjusted for age, sex, race, and proteinuria, the association remained strong and invariant across time-varying (aHR 0.30, 95% CI: 0.08–1.10), time-averaged (aHR 0.28, 95% CI: 0.07–1.07), and time-varying-cumulative (aHR 0.32, 95% CI: 0.08–1.21) proteinuria metrics.

**Conclusions:**

DAAs therapy was associated with improved graft survival and reduced mortality. While not statistically significant, the association was strong, and these single-center findings warrant larger studies to demonstrate the benefits of HCV treatment in this population.

Direct acting antiviral therapy is associated with decreased long-term graft failure and patient mortality in hepatitis c-infected kidney transplant recipients.

## INTRODUCTION

Despite the advent of direct-acting antiviral (DAA) therapy for the Hepatitis C virus (HCV), 71 million people remain infected worldwide with as many as 2.4 million chronically infected living in the United States (U.S.) [1]. While prevalence of infection in the U.S. may have declined in recent years, acute HCV cases tripled between 2010 and 2016 and the virus remains a serious public health concern [2-6]. HCV is considered a risk factor for numerous extrahepatic manifestations including proteinuria, chronic kidney disease (CKD) progression, and worse outcomes in kidney transplant recipients [7-15].

With emerging robust evidence of the major risks of complications for HCV-infected kidney transplant candidates and recipients, DAAs provide a significant opportunity to reduce the burden of HCV-related hepatic and extrahepatic complications in these patients and improve clinical outcomes [16]. We have recently observed short-term improvement in subclinical urinary protein/creatinine (P/C) ratios in association with DAA therapy in a cohort of kidney transplant recipients, suggesting that DAA utilization could be linked to improvements in kidney graft and patient outcomes [17]. Similarly, a recent study observed significant improvement in patient and graft survival rates in the short-term (1 year post-transplant) during the DAA era, among a nationally representative cohort from the United Network for Organ Sharing (UNOS) Registry [18]. Although this implied that a significant proportion of kidney transplant patients in the DAA era have received HCV therapy, this important study was limited by its inability to ascertain which of the HCV-infected patients completed DAA treatment and achieved cure. Additionally, data regarding the long-term effects of viral clearance on graft failure and mortality in this population remains unknown. The correct longitudinal metrics that we should utilize to monitor proteinuria trends in order to predict which patient subgroups are at greater risk of graft disease progression remains unclear [19,20]. In this study, we examined the association of DAA-induced cure with graft failure or death in kidney transplant recipients infected with HCV, and the strength of this association under specific longitudinal proteinuria metrics. Our intent was to compare outcomes in a group of DAA-treated patients with those of a historical, untreated comparison group. As access to HCV treatment expands in the DAA era, many untreated patients can still be found in the pre-DAA era, when very few qualified for interferon-based therapies.

## METHODS

This is a retrospective cohort follow-up study of patients seen at the University of Alabama at Birmingham (UAB) 1917 Viral Hepatitis Clinic and the UAB Liver Center between January 2007 and December 2015. We queried the electronic medical record (EMR) for all patients transplanted at UAB during the study period who had a positive HCV antibody at the time of transplant. The initial query produced 99 patients. We excluded patients if they had spontaneously cleared the virus (positive antibody but negative HCV viral load), were already lost to follow-up at the start of the study period, had transferred care to a different institution, or whose records were insufficient due to lack of routine urinary P/C ratio checks at clinic visits. After accounting for these exclusions, we identified 59 unique HCV-viremic kidney transplant recipients during this time frame. These patients were further divided into two cohorts: those who received DAA therapy and cleared the virus, and those who never received DAA therapy and remained persistently infected. The decision to treat with DAA was made in accordance with standard of care at the time patients were seen in the clinic. Patients were followed until December 2017. Data from routine clinical care were collected and extracted via chart screening using the UAB EMR (Cerner, Kansas City, MO). This study was reviewed and approved by the UAB institutional review board (protocol No. X150312011).

The events of interest were graft failure and death. Graft failure was defined as an estimated glomerular filtration rate (eGFR) <15 mL/min or resumption of dialysis. eGFR was calculated using the Chronic Kidney Disease Epidemiology Collaboration (CKD-EPI) formula, which utilizes age, sex, race and serum creatinine. [21]

The independent variable of interest was use of DAA therapy. DAA administration was confirmed by referencing the EMR, and all included patients completed full courses of DAA therapy as determined by standard of care.

We extracted data for age, sex, race, allograft donor status (deceased donor, living unrelated donor, living related donor), history of cirrhosis, viral genotype, baseline urinary protein/creatinine (P/C) ratios, P/C ratios over time, history of angiotensin converting enzyme inhibitor (ACEi) and angiotensin II receptor blocker (ARB) use, graft failure, and death. Baseline values were defined as the earliest post-transplant values available in the EMR for each patient. HCV status was confirmed by viral load and, if available, genotype. HCV cure was defined as sustained virologic response (undetectable viral load) at 12 weeks after completion of therapy (SVR12).

For each patient, we recorded all urinary P/C ratios collected routinely from date of transplant to the end of the study period. We utilized these P/C ratios to calculate longitudinal proteinuria metrics. The metrics were calculated as time-varying (TV), time-averaged (TA), and time-varying-cumulative (TVC) proteinuria [19]. TV proteinuria consisted of the instantaneous P/C ratios at each time point obtained during routine care. TA proteinuria was calculated as the mean P/C ratio value over the entire follow-up period. We calculated TVC using the trapezoidal method to determine the area under the curve when TV values were plotted against time. TV represents the P/C ratio at any given time during the follow-up period, TA represents the average of all TV values during the follow-up period, and TVC represents the cumulative proteinuria burden over time [19].

## STATISTICAL ANALYSIS

The event of interest was graft failure/death and the exposure of interest was DAA therapy. A priori calculations estimated that a sample size of 60 with an event rate of 25% would have 80% power to detect a statistically significant (alpha=0.05) hazard ratio of 0.22 or stronger in univariate analysis and 0.25 or stronger in multivariate analysis (r-square=0.10) for a variable with standard deviation (SD)=0.50.

Descriptive statistics included continuous and categorical variables. Continuous variables were reported as means (with standard deviations) when the distribution was “normal” and as medians (with quartiles: Q1=first quartile, Q3=third quartile) for “non-normal/skewed” distributions. Continuous variables were compared between the groups (eg, DAA vs non-DAA) by unpaired t-test or Wilcoxon rank-sum test as appropriate.

Categorical variables were reported as frequencies (with percentages) and compared by chi-square/Fisher's exact test.

Time from the transplant date to graft failure/death (event of interest) was evaluated using Kaplan-Meier survival curves. Median durability time was reported in years and compared across a stratified variable (DAA vs non-DAA) using the log-rank test examining statistically significant differences. The follow-up began on the date of transplant. Patients were followed until they experienced any event (graft failure/death), until study end (December 2017), or until they were lost to follow-up (last contact >18 months from study end), whichever was earliest. Those not experiencing an event were “censored.”

Association of various characteristics with the event was examined by univariate and multivariate Cox proportional hazard (PH) analyses and reported as crude and adjusted hazard ratios (cHR and aHR), respectively, with 95% confidence intervals (CIs). PH assumption for a categorical variable (eg, DAA) was examined both by entering the interaction (product) term of the variable and the natural logarithm of time in the models and by Schoenfeld residuals. PH assumption of a continuous variable (eg, TA) was examined using Martingale residuals. No evidence of deviation from the PH assumption was observed. The following clinically important confounders were included in multivariate analyses with DAA: proteinuria metrics, age, sex, and race. Three separate models were constructed for each of the proteinuria metrics (TV, TA, TVC).

To examine the robustness of the results, we performed four sensitivity/bias analyses (SA). First (SA #1), we assumed that all the patients lost to follow-up (LFU) had experienced an event, and we performed both univariate and multivariate analyses. Second (SA #2), we calculated the recently introduced E-value that represents the minimum strength of association that an unmeasured (or unknown/excluded from the model) confounder would need to have with both exposure and outcome (event) to fully explain a specific exposure-outcome association, conditional on the measured (or included in the model) covariates [22]. The advantages of E-value are that it does not require the specification of prevalence of unmeasured confounders or make assumptions about their nature, and its point estimate does not depend upon sample size but rather on the magnitude of the association. As the non-DAA group was somewhat more likely to undergo transplant in earlier years of the study period and, therefore, was a potential source of bias toward more events, we performed two additional sensitivity analyses (SA#3 and SA #4). In SA #3, we restricted the duration of follow-up to either 4 or 5 years. That is, the events that occurred after 4 or 5 years were “censored,” and the participants were reclassified depending upon whether they had received treatment or not within that period. Two separate Cox regression analyses (primary and LFU) were conducted for each of the three proteinuria metrics. In SA #4, we stratified the periods as 2007-2009, 2010-2012, and 2013-2015 and performed regression within each period, although this analysis did have the limitation of smaller sample size. Additionally, we constructed 95% CIs around the point estimates obtained in the primary analysis using the bootstrap method (100 replications).

Statistical significance was set at 0.05 (two-tailed). Sample size calculations were performed using PASS software, version 11 (NCSS). All other analyses were performed using SAS statistical soft-ware, version 9.4 (SAS Institute, Cary, NC).

## RESULTS

**Figure 1.**
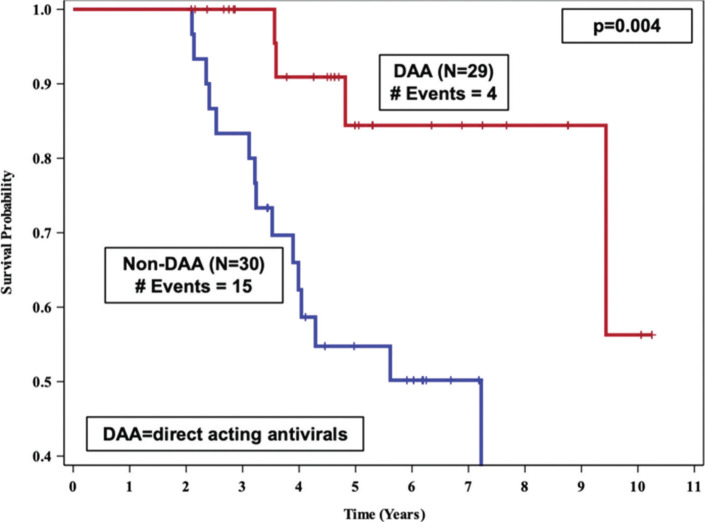
Kaplan-Meier survival curves comparing time to graft failure/death between DAA vs. non-DAA kidney transplant recipients at the University of Alabama at Birmingham, 2007-2017.

The overall analysis included 59 patients: 29 were included in the DAA group and 30 in the non-DAA group. Mean age was 55 years, 73% were African-American, 27% were Caucasian, and 68% were male. All patients were HCV-infected prior to kidney transplant. Nearly all patients in the DAA group received a sofosbuvir-based combination regimen: 21 combined with ledipasvir, 2 with simeprevir, 1 with velpatasvir, 1 with velpatasvir/voxipaprevir, 1 with ledipasvir plus ribavirin, and 2 with ribavirin. One patient received a sofosbuvir-free regimen (glecaprevir/pibrentasvir combination). Baseline median creatinine and P/C ratio were 1.4 mg/dL (Q1, Q3: 1.0, 1.6) and 0.5 (Q1, Q3: 0.3, 0.8) respectively. Baseline eGFR was 59 mL/min (Q1, Q3: 50, 74). No significant differences were observed with regard to demographics or other clinical characteristics between those who received DAA versus those who did not (Table 1). Median (descriptive statistics) post-transplant follow-up time was similar in both groups: 4.7 years for the DAA group vs 4.2 years for the non-DAA group (minimum follow-up: 2 years for both). Five patients were lost to follow-up in the non-DAA group and 1 in the DAA group (median follow-up=4.2). Median time to graft failure/death (survival curves, Figure 1) for those receiving DAA was undefined (did not reach 50%), while for the non-DAA group it was 7.2 years (*P*=0.004).

**Table 1.** Baseline Characteristics of Patients Undergoing Kidney Transplant at the University of Alabama at Birmingham, 2007-2015.

Characteristic	Total	DAA	Non-DAA	*P*-value
	N=59	n=29	n=30	
Event, n (%)				—
Graft failure	11 (19)	2 (7)	9 (30)	
Death	8 (13)	2 (7)	6 (20)	
None	40 (68)	25 (86)	15 (50)	
Age (as of transplant), years, mean (SD)	55 (8)	56 (9)	54 (8)	0.47^a^
Sex, n (%)				0.85^b^
Male	40 (68)	20 (69)	20 (67)	
Female	19 (32)	9 (31)	10 (33)	
Race, n (%)				0.27^c^
African American	43 (73)	23 (79)	20 (67)	
Caucasian	16 (27)	6 (21)	10 (33)	
Serum creatinine (mg/dL), median (Q1, Q3)	1.4 (1.0, 1.6)	1.5 (1.2, 1.6)	1.3 (1.0, 1.7)	0.60^d^
Protein:Creatinine ratio, median (Q1, Q3)	0.5 (0.3, 0.8)	0.6 (0.4, 0.8)	0.4 (0.2, 0.9)	0.23^d^
eGFR^e^ (mL/min/1.73m^2^), median (Q1, Q3)	59 (50, 74)	59 (54, 68)	61 (44, 89)	0.96^d^
Donor Type, n (%)				1.00^c^
Deceased	49 (83)	24 (83)	25 (83)	
Living unrelated	6 (10)	3 (10)	3 (10)	
Living related	4 (7)	2 (7)	2 (7)	
Taking ACEi/ARB, n (%)	35 (59)	20 (69)	15 (50)	0.70^b^
Year of transplant				<0.01^b^
2007–2009	16	5 (17)	11 (37)	
2010–2012	23	8 (28)	15 (50)	
2013–2015	20	16 (55)	4 (13)	
Post-transplant follow-up time (years), median (Q1, Q3)	4.6 (3.2, 6.3)	4.7 (3.6, 6.9)	4.2 (3.2, 6.2)	0.53^d^

ACEi=angiotensin-converting enzyme inhibitor; ARB=angiotensin II receptor blocker; DAA=direct acting antiviral; eGFR=estimated glomerular filtration rate; Q1=first quartile; Q3=third quartile; SD=standard deviation.

NOTE: Percentages have been rounded.

a Unpaired t-test.

b Chi-square test.

c Fisher's exact test.

d Wilcoxon rank-sum test.

e Calculated with the Chronic Kidney Disease Epidemiology Collaboration (CKD-EPI) formula which utilizes age, sex, and race.[20]

Overall, 29 patients (49%) received DAA while the remaining 30 (51%) did not receive DAA and remained persistently infected. Of those who received DAA, 24 (83%) achieved cure (SVR12) by censoring date. Among the remaining 5 DAA patients, 1 had documented SVR12 after censoring, and 4 did not have documented evidence of SVR12. For those who received DAA therapy, median time from kidney transplant to DAA was 3 years (Q1, Q3: 1.1, 4.5) and was similar in those with versus those without event (3.1 vs 3.0 years); none of the patients received DAA prior to transplant. From the start of the follow-up period to the time of cohort censoring in 2017, none of the historical non-DAA patients subsequently crossed over and received DAA therapy. Overall, 19 patients (32%) experienced an event during follow-up; the DAA group was lower (4/29=14%) than the non-DAA group (15/30=50%). In the DAA group, there were 2 graft failures and 2 deaths, while in the non-DAA group there were 9 graft failures and 6 deaths. Follow-up in treated patients was 152.2 person-years, and in untreated patients it was 145.8 person-years. We observed 4 and 15 events in the DAA and non-DAA groups, respectively; the incidence rates were 2.6 and 10.3 per 100 person-years (*P*=0.02) with an incidence rate ratio of 0.26 (95% CI: 0.08–0.77). Stratification of patients by period of transplantation showed lower rate of events in the DAA group: Year 2007-2009 (1/5[(20%] vs 4/11 [36%]); Year 2010-2012 (1/8 [13%] vs 9/15 [60%]); and Year 2013-2015 (2/16 [13%] vs 2/4 [50%]). The causes of death (N=8) included sepsis (n=5), traumatic subdural hemorrhage (n=1), and unclear reasons not captured in the UAB EMR (n=2). Among those who died, 4 deaths were preceded by graft failure, nephrotic syndrome, or signifi-cant decline in GFRs; 5 had kidney graft biopsy information available (1 with negative biopsy for acute rejection; 1 with acute rejection and chronic transplant glomerulopathy, 1 with tubulitis, interstitial fibrosis, and membranous proliferative glomerulopathy likely due to HCV; and 2 with borderline rejection). Among 2 patients with unclear cause of death, 1 had *Haemophilus influenzae* sepsis 5 months prior to death and the other patient had preserved graft function until being lost to follow-up in 2016. This patient re-presented with graft failure and hemodialysis in April 2017, 2 months prior to death.

In univariate analysis, viral clearance using DAAs was strongly associated with lower risk of an event (cHR=0.19, 95% CI: 0.06–0.66; *P*=0.01) (Table 2). In multivariate analyses, when adjusted for baseline characteristics such as age, sex, race, and proteinuria, this association remained strong and invariant across the proteinuria metrics, although not statistically significant at a level of 0.05: TV: aHR=0.30 (95% CI: 0.08–1.10; *P*=0.07); TA: aHR=0.28 (95% CI: 0.07–1.07; *P*=0.06); TVC: aHR=0.32 (95% CI: 0.08–1.21; *P*=0.09) (Table 2).

**Table 2.** Cox Proportional Hazard Regression Analyses Examining Characteristics Associated With Graft Failure or Death in Kidney Transplant Recipients at the University of Alabama at Birmingham, 2007-2017.

Characteristic	Univariate analysis		Multivariate analyses (N=59)					
	Crude HR (95% CI)	*P*-value	Adjusted HR (95% CI)	p-value	Adjusted HR (95% CI)	p-value	Adjusted HR (95% CI)	*P*-value
**A. Outcome of interest**								
Group: DAA vs Non-DAA^a^	0.19 (0.06 – 0.66)	0.01	0.30 (0.08 – 1.10)	0.07	0.28 (0.07 – 1.07)	0.06	0.32 (0.08 – 1.21)	0.09
**B. Confounders**								
Age (per 1-year increase)	0.98 (0.92 – 1.04)	0.51	0.97 (0.90 – 1.05)	0.49	0.97 (0.90 – 1.04)	0.40	0.98 (0.91 – 1.06)	0.58
Sex: Male vs Female^a^	1.16 (0.41 – 3.29)	0.78	1.46 (0.42 – 5.14)	0.56	1.31 (0.38 – 4.47)	0.67	1.47 (0.41 – 5.29)	0.56
Race: African American vs White^a^	0.69 (0.26 – 1.83)	0.46	0.78 (0.24 – 2.54)	0.68	0.81 (0.26 – 2.50)	0.72	0.66 (0.20 – 2.18)	0.49
Proteinuria Metrics (per unit increase)								
Time-varying	1.51 (1.27 – 1.80)	<0.001	1.40 (1.16 – 1.70)	0.001	--	--	--	--
Time-averaged	2.16 (1.40 – 3.31)	0.001	--	--	1.69 (1.04 – 2.74)	0.04	--	--
Time-varying cumulative	2.54 (1.67 – 3.87)	<0.001	--	--	--	--	2.05 (1.28 – 3.29)	0.003

CI=confidence interval; DAA=direct acting antiviral; HR=hazard ratio; P/C: Protein/Creatinine.

a Reference category.

In sensitivity/bias analyses (SA #1), when all the 6 LFU patients were regarded as having had an event, the associations remained strong and statistically significant. In univariate analysis, the cHR was 0.19 (95% CI: 0.07–0.56; *P*=0.003). In multivariate analyses, the associations remained invariant across the proteinuria metrics: TV: aHR=0.25 (95% CI: 0.08–0.77; *P*=0.02); TA: aHR=0.24 (95% CI: 0.08–0.76; *P*=0.02); TVC: aHR=0.27 (95% CI: 0.09 – 0.87; *P*=0.03) (full models not shown). The E-value (SA #2) for multivariate analyses ranged from 5.7 to 6.6 depending upon the proteinuria metric. In other words, a strong association would have been needed between the (unmeasured/unknown/excluded) confounders and the event to explain away (ie, HR=1 meaning no association) the observed associations. In bootstrap analysis, the 95% CI for the association of DAA within TV, TA, and TVC were: 0.00–1.10, 0.00–1.22, and 0.00–1.22 respectively, in multivariate analysis.

The non-DAA group was somewhat more likely to undergo transplant in the earlier years of the study period (Table 1), a potential source of bias towards more events. However, among those who did not have events, the follow-up duration did not differ significantly between the DAA and non-DAA groups (4.7 years vs 6.2 years, *P*=0.20; both with a minimum duration of 2 years and a maximum duration of 9–10 years). Furthermore, in SA #3 analyses, where follow-up was restricted to 4 or 5 years, DAA treatment remained associated with lower risk of the event across the TV, TA, and TVC proteinuria metrics (aHR=0.31, 0.31, and 0.32 at 4 years; and aHR=0.37, 0.36, and 0.36 at 5 years, respectively) (Table 3A). This relationship remained the same when LFU patients were counted as events (Table 3B). Similarly, in SA #4 (regression in stratified periods) this relationship remained the same (models not shown).

**Table 3.** Cox Proportional Hazard Multivariate Regression Analyses Examining Association of Treatment With DAA With Graft Failure or Death in Kidney Transplant Recipients at the University of Alabama at Birmingham, 2007-2017 (Sensitivity analysis #3).

P/C ratio	Follow-up restricted to			
	4 years		5 years	
	Adjusteda HR (95% CI)	P-value	Adjusteda HR (95% CI)	P-value
**A. Primary analysis**				
TV	0.31 (0.06 – 1.50)	0.14	0.37 (0.10 – 1.39)	0.14
TA	0.31 (0.07 – 1.44)	0.14	0.36 (0.10 – 1.37)	0.13
TVC	0.32 (0.06 – 1.61)	0.17	0.36 (0.09 – 1.41)	0.14
**B. Lost to follow-up patients (n = 6) counted as events**				
TV	0.25 (0.07 – 0.92)	0.04	0.30 (0.10 – 0.93)	0.04
TA	0.25 (0.07 – 0.87)	0.03	0.29 (0.09 – 0.91)	0.03
TVC	0.25 (0.07 – 0.94)	0.04	0.30 (0.09 – 0.95)	0.04

CI=confidence interval; DAA=direct acting antiviral; HR=hazard ratio; P/C: Protein/Creatinine; TV=time-varying; TA=time-averaged; TVC=time-varying cumulative.

a Adjusted for age as of transplant, sex, race, P/C ratio.

NOTE: The adjusted HR are for the DAA vs Non-DAA (reference category) across the three proteinuria metrics.

## DISCUSSION

We examined the long-term association of proteinuria trends and DAA therapy with graft failure and mortality in HCV-infected kidney transplant patients from a single center. Incidence of graft failure and death was significantly lower in the long term in patients who received DAA therapy. We found that DAA therapy was strongly associated with lower risk of graft failure or death in univariate analysis. In multivariate analyses that accounted for age, sex, race, and proteinuria, the independent association between DAA therapy and lower risk of graft failure and death trended towards statistical significance.

There is extensive evidence for the morbidity and mortality associated with HCV infection in kidney transplant recipients [7-15]. As the use of DAA therapy becomes more common, there is a need to further understand the effects of HCV viral clearance on outcomes in renal transplant patients. Sawinski and Bloom discuss the deleterious effects of HCV infection on renal transplant recipients, such as increased risk of glomerular disease and new-onset diabetes, and the hope for improved outcomes with the advent of DAA therapy, while also acknowledging the paucity of studies examining outcomes after viral clearance in the DAA era [16]. Sabbatini and colleagues have studied the effects of HCV eradication in renal transplant patients on subjective measures such as emotional domains and quality of life. In their small prospective cohort study, they found that chronic viral infection represented a substantial psychological burden. Viral clearance was associated with significant improvement in quality of life, but this study did not examine objective outcomes such as graft or patient survival, rejection rates, or transplant complications [23].

We have previously reported short-term improvement in subclinical proteinuria in kidney transplant patients after DAA cure, suggesting potential for improved graft outcomes with viral clearance, as proteinuria is a surrogate for graft pathology [7-9,12,17,19,20]. Expanding on our preliminary observations, Wong and colleagues examined short-term graft and patient outcomes during the DAA era. In the UNOS registry, these investigators examined short-term patient and graft survival for 62,655 patients (4,701 of whom were HCV+), and observed improvement in patient and graft survival at 1 year post-transplant during the DAA era. In this large dataset, they were unable to ascertain DAA therapy receipt and SVR12 status of patients. However, compelling improvements in patient and graft survival were observed after the advent of DAAs, enough to close the survival gap between the HCV-infected cohort and the uninfected one [18]. Our data reported here build on these findings by examining graft and patient outcomes in the long term after HCV eradication with DAAs, with median post-transplant follow-up time of 4.5 years. In our study, DAA therapy was independently associated with trends toward improved graft survival and reduced mortality in the long term. The association was strong, nearly reaching the threshold for statistical significance. We observed a substantial difference in frequency of events between the treated and untreated cohorts, with patients who remained persistently infected with HCV experiencing a higher incidence of graft failure and death during the study follow-up period. The observations from our cohort, albeit in a small sample size, support the notion that the survival benefit observed in the UNOS registry at 1 year may be durable and sustained in the long term [17,18]. These findings support the well-documented association of HCV infection with morbidity and mortality among kidney transplant recipients, suggesting that treating HCV and achieving viral eradication should be a priority in this population [7-17].

HCV infection contributes to morbidity and mortality through several mechanisms. Kidney-specific mechanisms include glomerular immune complex deposition (eg, cryoglobulinemia), direct renal parenchymal invasion, and renal complications of extrarenal manifestations [24-26]. HCV is also known to cause direct endothelial damage, accelerate atherosclerosis, and contribute to increased cardiovascular mortality [27]. And, of course, HCV contributes to liver-related morbidity and mortality, namely cirrhosis, hepatocellular carcinoma, and their associated complications. Adinolfi and colleagues have demonstrated improvement in atherosclerosis with HCV viral clearance with DAAs [27]. Several other studies have shown improvements in inflammatory biomarkers and cryoglobulinemia with DAA therapy [28-31]. It stands to reason that viral clearance with DAAs would mitigate these numerous deleterious consequences of HCV infection and potentially promote improved graft and patient outcomes.

The growing body of evidence for successful HCV treatment and improved outcomes in kidney transplant recipients could have implications for expanding the donor pool to include HCV-infected donors for HCV-negative recipients [32]. While it appears that graft and patient mortality risk decrease after viral clearance, it remains unclear whether mortality risk returns to baseline or an intermediate level of risk relative to patients who have never been infected [33]. Future work should address these questions. We also expect that larger studies will further elucidate the treatment effect seen in our cohort as access to DAAs, the current standard of care, increases.

Our study results should be interpreted cautiously in lieu of limitations such as small sample size, retrospective design, and possible unmeasured confounding. For example, we were unable to control for immunological incompatibility risk, behavioral factors such as engagement in care, and variations in clinician practices, although we expect these differences to be minimized in patients who showed eligibility for multifaceted post-kidney transplant care. Despite these limitations, Table 1 shows that the baseline cohort characteristics are reasonably equivalent and comparable between the two groups, indicating a balance of confounders. Furthermore, sensitivity/bias analyses indicated that the results were robust, and could have been stronger and statistically significant at the 0.05 level had there been more events among those patients who were lost to follow-up (SA #1). Thus, the observed associations reported in this study would tend to underestimate the true effect of DAA therapy. Moreover, as indicated by the high E-values (independent of sample size), a strong association of unmeasured/unknown/excluded confounders would have been needed to explain away the observed associations (SA #2). Also, SA #3 and SA#4, which adjusted for potential bias in longer follow-up of the non-DAA group, showed that the direction of the association remained the same, indicating robustness of the results. Due to small sample size, our results had wide confidence intervals and did not reach statistical significance, but the association remained strong in multivariate and sensitivity/bias analyses. Lack of statistical significance should not be equated with lack of any association, statistical significance being dependent upon sample size [34].

Our study included 29 patients who underwent DAA therapy and would certainly benefit from higher HCV treatment uptake in the early years of DAA adoption (2014-2016). While it's unclear why some patients in this era did not undergo DAA therapy, we can speculate on several potential reasons. In the early years of the DAA era, treatment cost was extremely high, and access was significantly limited by payors. The prior authorization approval process was complicated and lengthy, which potentially reduced the number of patients applying for and being approved for therapy at our single study site. Additionally, other competing priorities in these complex patients (including lack of experience in DAA use among kidney transplant populations and concerns about drug-drug interactions) may have delayed prompt referral and treatment scale-up. Lastly, our study does not assess differences in frequency of acute graft failure, especially with reports of variations in tacrolimus levels that may occur during or after HCV treatment.

## CONCLUSION

DAA cure was associated with a strong, independent (although not statistically significant) benefit in long-term graft failure and patient mortality. The observed associations between subclinical longitudinal proteinuria metrics and survival suggest that clinically silent and unchecked HCV replication may need prompt attention early in the post-transplant period. These findings warrant larger prospective studies to demonstrate the benefits of HCV treatment in renal transplant recipients in the long term.
